# Impact of the Type of Crosslinking Agents on the Properties of Modified Sodium Alginate/Poly(vinyl Alcohol) Hydrogels

**DOI:** 10.3390/molecules26082381

**Published:** 2021-04-19

**Authors:** Katarzyna Bialik-Wąs, Ewelina Królicka, Dagmara Malina

**Affiliations:** 1 Department of Organic Chemistry and Technology, Faculty of Chemical Engineering and Technology, Cracow University of Technology, 24 Warszawska St., 31-155 Cracow, Poland; ewelinakrolicka@gmail.com; 2 Department of Chemical Technology and Environmental Analytics, Faculty of Chemical Engineering and Technology, Cracow University of Technology, 24 Warszawska St., 31-155 Cracow, Poland; dagmara.malina@pk.edu.pl

**Keywords:** SA/PVA hydrogels, *Echinacea purpurea*, release profile, various crosslinking agents

## Abstract

Here, we report on studies on the influence of different crosslinking methods (ionic and chemical) on the physicochemical (swelling ability and degradation in simulated body fluids), structural (FT-IR spectra analysis) and morphological (SEM analysis) properties of SA/PVA hydrogels containing active substances of natural origin. First, an aqueous extract of *Echinacea purpurea* was prepared using a Soxhlet apparatus. Next, a series of modified SA/PVA-based hydrogels were obtained through the chemical crosslinking method using poly(ethylene glycol) diacrylate (PEGDA, M_n_ = 700 g/mol) as a crosslinking agent and, additionally, the ionic reaction in the presence of a 5% *w*/*v* calcium chloride solution. The compositions of SA/PVA/*E. purpurea*-based hydrogels contained a polymer of natural origin—sodium alginate (SA, 1.5% solution)—and a synthetic polymer—poly(vinyl alcohol) (PVA, M_n_ = 72,000 g/mol, 10% solution)—in the ratio 2:1, and different amounts of the aqueous extract of *E. purpurea*—5, 10, 15 or 20% (*v*/*v*). Additionally, the release behavior of echinacoside from the polymeric matrix was evaluated in phosphate-buffered saline (PBS) at 37 °C. The results indicate that the type of the crosslinking method has a direct impact on the release profile. Consequently, it is possible to design a system that delivers an active substance in a way that depends on the application.

## 1. Introduction

From a medical, pharmaceutical and biomedical point of view, hydrogels are a very important group of polymeric materials. They constitute a three-dimensional hydrophilic network of crosslinked polymeric chains [1,2,3]. Their main feature is the ability to absorb (swell) and retain water inside the macromolecule without dissolving it. Polymer chains in a dry state are in the form of tightly closed “bundles”, while under the influence of water molecules, the functional groups present in the chains are solvated and dissociated [1,4,5].

Furthermore, hydrogel matrices are an indispensable class of materials for biomedical applications due to their high biocompatibility, the ability to absorb water and other fluids in a reversible manner, as well as good mechanical properties within biological tissue [2,4,6]. The water-sorption capacity is attributed to the interaction between water molecules and polar groups, which are present in the polymer matrix. In addition, their chemical structure allows gradual drug release, which is associated with the prolongation of its action. As far as the role of hydrogel matrices in the medical field is concerned, an important application is wound dressings, which can be used in the treatment of burn wounds, pressure ulcers as well as post-operative wounds. Moreover, hydrogel matrices are used in the production of contact lenses, in tissue engineering and as a controlled drug release system [2,7,8,9,10]. Currently, hydrogels based on natural polymers, such as polysaccharides and proteins, are very popular and desirable. From the point of view of medical and pharmaceutical applications, alginates are very promising, because they belong to biocompatible and biodegradable components. In our research, we used alginates, which constitute linear polymers composed of (1→4)-α-L-guluronic acid blocks (GG), (1→4)-β-D-mannuronic acid blocks (MM) and, additionally, heteropolymeric sequences of M and G (MG blocks) [11,12]. Moreover, in the case of the preparation of hydrogel or composite materials, alginates are used together with some additional polymers, such as: poly acrylamide (PAAm) [13], poly(acrylic acid) (PAA) [14], gelatin [15], and chitosan [16], as well as PVA [17]. Poly(vinyl alcohol) is very important and interesting, because it characterized by biocompatibility, biodegradability and non-toxicity due to it is commonly used in the medical and pharmaceutical, such as: artificial organs, drug delivery as well as wound dressings. Moreover, PVA hydrogels exhibit not only good biocompatibility, but also good physicochemical and especially outstanding bio-tribological properties [18,19,20,21].

It is worth noting that in recent years there has been a trend showing the interest of researchers in modifying hydrogels intended to wound healing also with medicinal substances of natural origin, mainly extracted from plants [22]. For example, it has been shown that *Cryphaea heteromalla* aqueous extract in the hydrogel film may prevent excess oxidative stress generation during wound healing [23]. In turn, Ciolacu et al. incorporated lignin into the hydrogel and produced hydrogel structures with a higher drug release rate; however, Wang and Chen successfully tested the influence of the addition of cellulose nanowhiskers to cellulose physical gels resulting in the more steady release of the protein. [24,25].

The introduction of additional active substances of natural origin, such as the *Echinacea purpurea* extract or *Aloe vera*, into the hydrogel composition allows the preparation of materials with improved healing properties. *Echinacea purpurea* contains many active substances, such as polysaccharides, caffeic acid derivatives (including cichoric acid), alkylamides, and glycoproteins [10,11]. The most active compounds of *Echinacea purpurea* are polyphenols—derivatives of caffeic acid: caftaric acid, chlorogenic acid, cynarin and silicic acid [12]. They play a significant role in the therapeutic effect of this plant, especially in activating the immune system by triggering the alternating complement pathway, as well as increasing the number of white blood cells distribution, stimulating phagocytosis, T-cell production, lymphocytic activity, cytokine production, cell respiration and enzyme secretion [26,27]. These bioactive ingredients are characterized by pharmacological antiviral, anti-inflammatory, bacteriostatic and immunoregulatory effects [28].

Generally, bio-based hydrogels can be prepared using several chemical and ionic crosslinking methods and also in different ways, e.g., photopolymerization and exposure to radiation (e.g., gamma rays or microwaves) [29]. The chemical method is based on the formation of covalent bridges during the reaction between the polymer or monomer with the crosslinking agent (N, N’-methylenebisacrylamine (NMBA) and poly(ethylene glycol) diacrylate (PEGDA) in the presence of an initiator (ammonium, potassium or sodium persulfates [30,31,32]. Meanwhile, the method of crosslinking by ionic interaction takes place in the presence of bivalent or multivalent ions, such as Ca^2+^, Cu^2+^, Fe^2+^ or Al^3+^. One of the best-known polymers that can be crosslinked by ionic interaction is alginates [33]. This group of polysaccharides can form a three-dimensional structure under the influence of bivalent ions at room temperature and in a physiological environment (pH = 5.2–5.5). This kind of gelation process is quite interesting, because the ionically crosslinked alginates make it possible to form a three-dimensional structure called the “egg box” model. Divalent ions are attached to guluronic acid by ionic bonding, and then the guluron block of one polymer chain connects with adjacent G blocks [33,34,35]. The chemical structure of alginate has a great influence on the properties of alginate hydrogels obtained by this method. Hydrogels made from M-block-rich alginate are very soft and brittle, and may also be less porous. On the other hand, gels obtained from alginates with a high content of G blocks show higher stiffness and porosity [35,36,37]. Comparing both methods, crosslinking of alginates by chemical method leads to obtaining hydrogels that are characterized by higher mechanical strength. The obtained hydrogels have an ordered, complex three-dimensional structure and a higher crosslinking density. In addition, they show greater stability in physiological solutions compared to hydrogels obtained by the ionic method. Ion-crosslinked alginate hydrogels lose more than 60% of their initial mechanical strength within 15 h of exposure to physiological buffers. The reason for this may be the ion exchange between divalent ions inside the hydrogel and monovalent ions contained in the surrounding fluid [9,38,39].

The basic principle of crosslinking with ultraviolet radiation is the conversion of UV radiation into chemical energy and can occur through the radical or ionic mechanism [40]. In the case of the radical mechanism, the unsaturated bonds present in a polymer chain are polymerized. The presence of photoinitiators, i.e., benzoin, dimethoxyacetophenone, acyloximeester or benzoyl ketals, is necessary for the initiation of the crosslinking reaction in the radical mechanism. On the other hand, crosslinking via the cationic mechanism consists in the opening of epoxy rings or the reaction of vinyl groups, which are present in the polymer chain [40,41,42]. Another method of obtaining hydrogel matrices is the application of radiation, which connects polymer chains by means of covalent bonds. They are not broken down and, as a result, the entire macromolecule is not destroyed. The initiator of the crosslinking process is gamma radiation coming from the isotope of cobalt ^60^Co or cesium ^137^Cs, and it can also be an electron beam from an accelerator. Radicals are formed on polymer chains following symmetric breaking of the C-H bond. This decay is caused by gamma radiation, which strikes polymer solutions and initiates the formation of radicals. Additionally, hydroxyl radicals that have an unpaired electron are formed [43,44].

In summary, in this article, the influence of the type of crosslinking agent on the properties of modified hydrogels is investigated. It turns out that different crosslinkers yield totally different changes in the properties of obtained final products. The method of crosslinking definitely affects the morphology of hydrogels as well as the physicochemical properties of the materials obtained for future dressings. In-depth knowledge about the behavior and properties allow to select the parameters of the production process and the method of crosslinking to design the release of active substances from the matrix in accordance with the expectations, and thus predict the direction of wound treatment. This means that the research presented in this study is up-to-date and allows to enrich the general knowledge about the possibilities of using hydrogel substances in medicine.

## 2. Results and Discussion

### 2.1. Swelling Abilities

**Figure 1:**
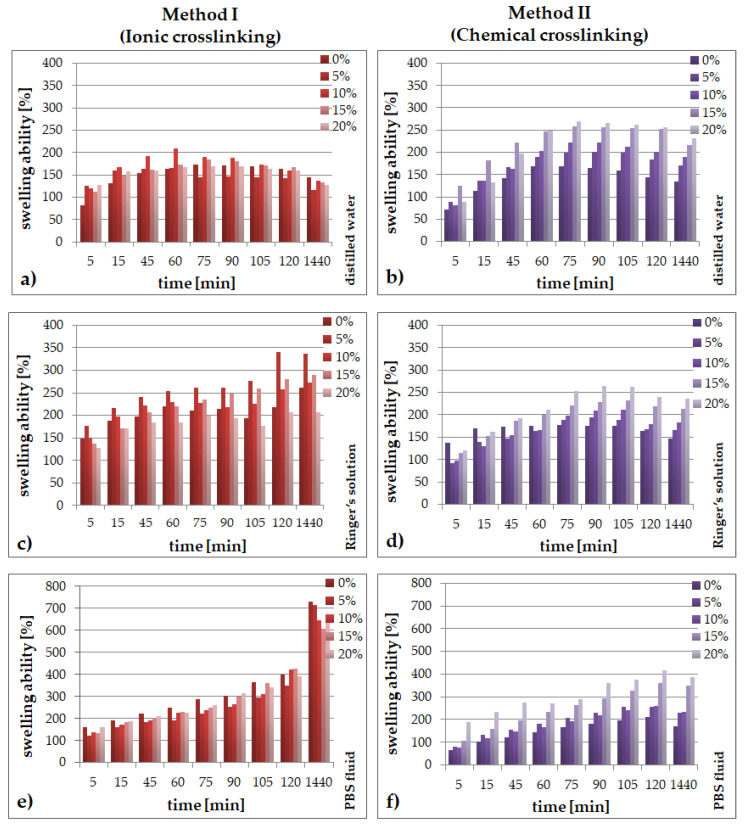
Swelling ability for SA/PVA/*EP* hydrogels obtained using method I (left side) or method II (right side) in: (**a**,**b**) distilled water; (**c**,**d**) Ringer’s solution; (**e**,**f**) in PBS fluid.

The results on the swelling abilities of SA/PVA/*EP* hydrogels obtained using: (a) ionic and (b) chemical crosslinking in the period from 5 to 1440 min (24 h) after immersion in distilled water, Ringer’s solution and PBS are presented in Figure 1.

The hydrogels prepared through ionic crosslinking were characterized by greater swelling abilities in comparison to the samples obtained via the chemical reactions. This is due to the fact that ionically crosslinked hydrogels contain sodium alginate, which in contact with Ca^2+^ ions undergoes additional crosslinking, which may result in inhibition of fluid absorption. This phenomenon may also result from the different structure, morphology and porosity of the materials. The chemically crosslinked hydrogels, in the environment of Ringer’s liquid and distilled water, did not stand out in terms of very high values of the swelling coefficient. This result may come from ion exchanges between the H^+^ ions present in the system and the metal ions present in the solutions. Comparing the media used in the study, the PBS liquid samples show greater stability and a more compact and flexible structure compared to the samples incubated in distilled water and Ringer’s solution.

Furthermore, in the case of the chemically crosslinked hydrogels, it was observed that with an increase of the amount of the extract, the swelling degree increases. Similar results were obtained in the previous studies of the authors [45]. The highest values were recorded for the introduction of 20% (*v*/*v*) of the additive. This is due to the fact that the packing density of the chains in the hydrogel matrix decreases with increasing amounts of additional components in the system. When an active ingredient, which is then released from the hydrogel matrix, is used at a higher concentration, it creates additional voids that can replace the absorbed fluid. Weakly crosslinked hydrogels tend to absorb more water and other fluids easily. On the other hand, a highly crosslinked structure cannot hold a large amount of water in the gel network [45,46,47,48].

Summarizing, the type of the crosslinking method had a strong influence on the ability of the materials to absorb simulated fluids. However, when the amount of the extract in the matrix increased, the swelling capacity resulting from the chain-packing loosening in the hydrogel matrix also increased, regardless of the crosslinking method used.

### 2.2. Degradation Tests

**Figure 2:**
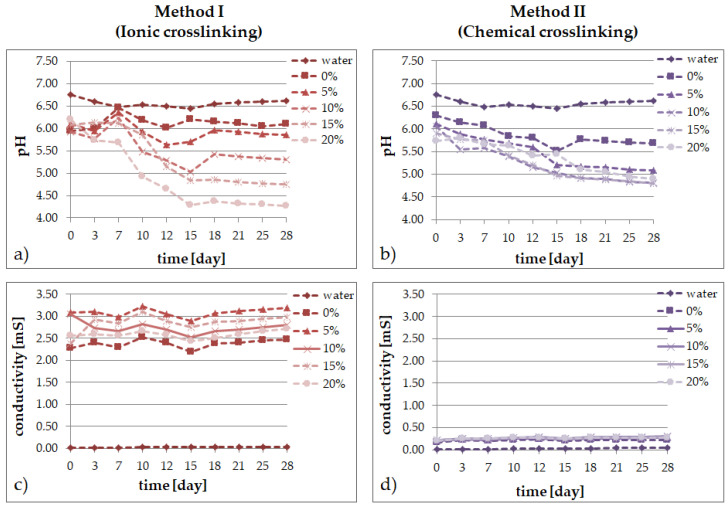
Changes of distilled water during 28 days for SA/PVA/*EP* hydrogels prepared using method I (left side) or method II (right side) in pH value (**a**,**b**) and conductivity (**c**,**d**).

**Figure 3:**
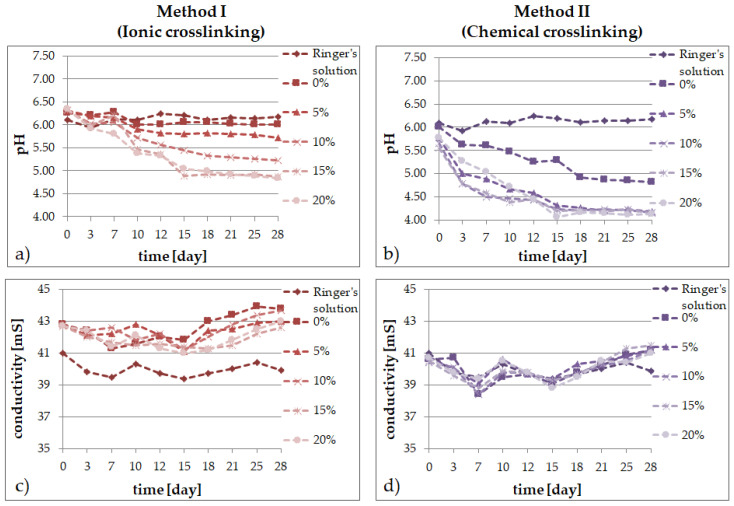
Changes of Ringer’s solution during 28 days for SA/PVA/*EP* hydrogels prepared using method I (left side) or method II (right side) in pH value (**a**,**b**) and conductivity (**c**,**d**).

**Figure 4:**
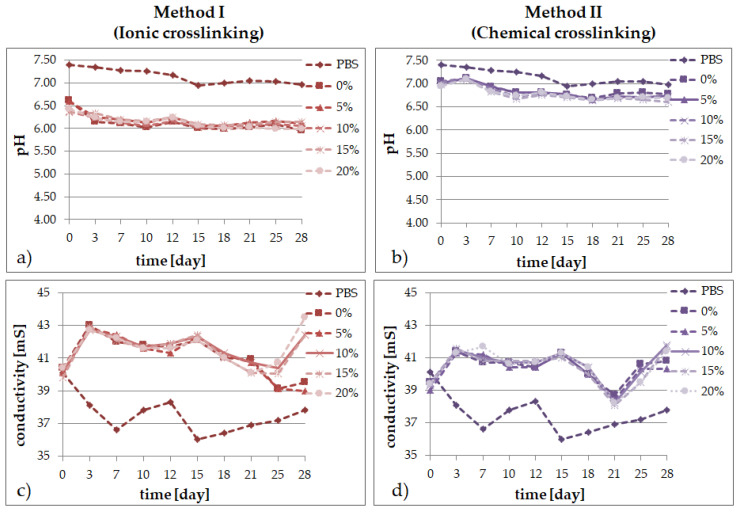
Changes of PBS fluid during 28 days for SA/PVA/*EP* hydrogels prepared using method I (left side) or method II (right side) in pH value (**a**,**b**) and conductivity (**c**,**d**).

In solutions simulating the living organism environment, the phenomenon of progressive degradation was followed for 28 days, which is illustrated graphically: Figure 2 in distilled water; Figure 3 in Ringer’s solution; and Figure 4 for degradation in PBS. The degradation of bioresorbable hydrogels is mainly related to the weight loss caused by the decomposition of polymers into macromolecular substances, as well as the breaking of hydrogen bonds and van der Waals interactions. Measurements of pH and conductivity taken during the 28-day incubation of samples show the effect of the hydrogel on the properties of the fluid.

The ionic inertia of distilled water affects the behavior of hydrogels in its environment. In the case of the materials obtained with method I, it can be observed that the content of the extract up to 5% (*v*/*v*) is a limit concentration that does not cause significant changes in the pH of the water during immersion. For higher contents, pH decreases significantly. The first measurements for the ionically crosslinked matrix show a slight increase in the water pH when the system pH tends to grow towards 6.5. From the 7th day, strong mass degradation was observed. Most likely, the decomposition products accumulated inside the material autocatalyzed the bio-eruption process, resulting in a pH drop. The chemically crosslinked hydrogels behave differently. In the first days, a slight decrease in pH is observed, which is compensated for by a gradual decrease in the following days and the stabilization of its value.

The presence of the *E. purpurea* extract in the materials incubated in water did not strongly affect the conductivity level. Similar results were obtained in the previous studies of the authors [45]. However, an addition of the extract caused a slight increase in the water conductivity at the beginning of incubation and then the conductivity is at a constant level throughout the entire experiment time. The incubation of analogous materials, but chemically crosslinked, also revealed a decrease in the pH value with an increasing extract content, but less rapid and proportionate. On the other hand, the conductivity in every sample remained close to the initial level throughout the incubation period—the materials incubated in water were rather stable in time.

The degradation in Ringer’s fluid occurs differently depending on the crosslinking method. For the ionically crosslinked materials, an increase in the pH value was observed even up to 7 days of incubation, whereas after about a 2-week-long incubation, stabilization of the pH value was seen. However, for the chemically crosslinked matrix, a decrease in pH was observed from the first days. As in the case of the analysis of materials degradation in water, also in Ringer’s solution, the decrease in pH over time depends on the extract content—a higher concentration of the additive leads to a lower pH value in time even by two units after more than two weeks of incubation for the samples containing the extract. When analyzing the level of the Ringer’s fluid conductivity over time, slight alternating increases and decreases of this parameter were observed in all the tested materials, modified with the extract and the control sample. The trends were similar for every sample, but the presence of the material in the fluid slightly increased the conductivity. On the other hand, the conductivity was lower in the materials obtained with method II and the trend of changes was similar in all the cases, regardless of the extract presence.

The PBS fluid turned out to be the most stable medium when assessing the degree of hydrogel matrices degradation in time. The materials obtained by the first method were stable throughout the incubation period, after the initial decrease in pH by 1 unit, the lower value was maintained for 28 days in all the variants. In the ionically crosslinked samples placed in the PBS solution, a slight increase in the pH value was observed in the first days of incubation. This may be related to the release of calcium chloride. In the following days, we observed a drop in pH caused by the presence of phosphate ions, which can create a slightly acidic environment.

The conductivity changes for the samples immersed in the PBS solution and Ringer’s fluid show a jump profile of ion release from the degraded matrix. In the first days, there is a drop in conductivity in the case of the Ringer’s fluid hydrogels. This may be due to delayed crosslinking of the material by absorbing calcium ions from the solution. In the following days, the value increases due to the hydrolysis of the hydroxyl and ester bonds. When analyzing the conductivity diagrams for water for the ionically crosslinked materials, we observe a slight increase in the value in the first days of incubation, which may be related to the presence of calcium ions in the hydrogel structure. This suggests that the method of matrix synthesis did not significantly affect the differences in the changes of the PBS fluid conductivity over time, the trends in both cases are similar. However, when the chemical crosslinking method is used, the conductivity of the fluid increases slightly during the entire incubation period.

Comparing the materials containing different amounts of the *Echinacea purpurea* extract, we observe that matrices with a higher extract content react more rapidly to environmental changes. A tendency can also be observed whereby over time, the pH of the solutions decreases and the conductivity increases. This is most likely due to the degradation of the polymer matrix, which leads to the formation of oligomers in the system and acidifies the environment.

### 2.3. FT-IR Analysis

**Figure 5:**
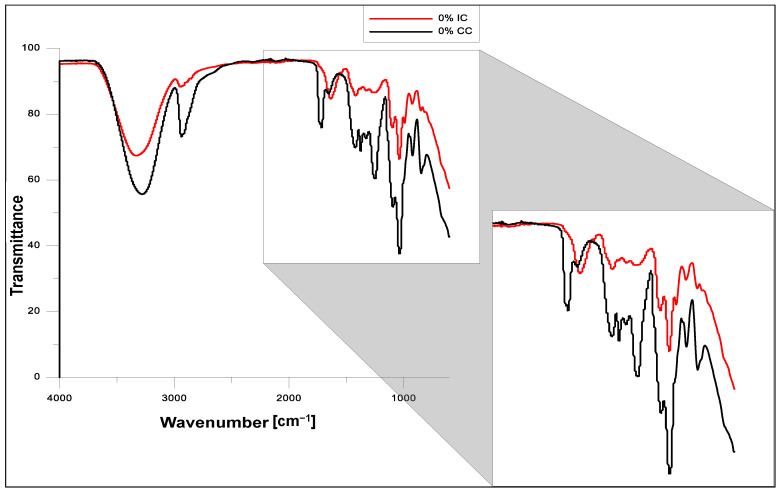
FT-IR spectra of SA/PVA hydrogels prepared using method I (ionic crosslinking, IC) or method II (chemical crosslinking, CC).

**Figure 6:**
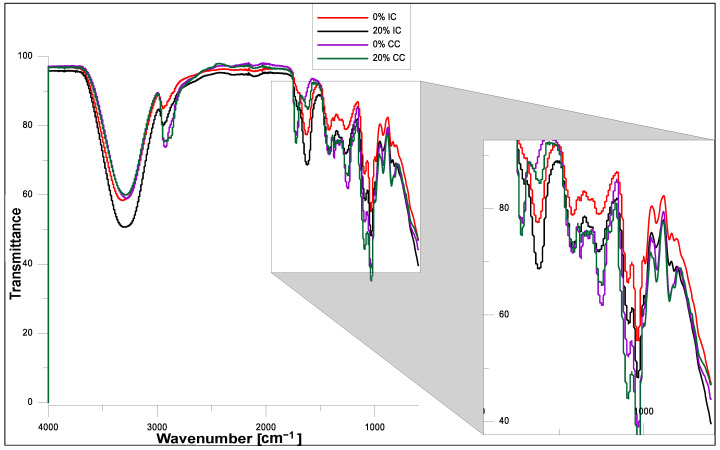
FT-IR spectra of SA/PVA hydrogels modified by *Echinacea purpurea* extract prepared using method I (ionic crosslinking, IC) or method II (chemical crosslinking, CC).

The FT–IR spectra of the hydrogels obtained using ionic or chemical crosslinking before and after modification with the *Echinacea purpurea* extract are presented in Figure 5 and Figure 6, respectively. The FT-IR analysis confirmed the presence of characteristic functional groups of the polymeric matrices and showed that the presence of the plant extract does not affect significantly the chemical structure of the modified hydrogels, which was also confirmed in our other, already published paper [45,49]. However, it turns out that the *Echinacea purpurea* extract as well as a type of crosslinkers impact directly on the crosslinking of the hydrogel matrix.

In the wave number range of 3000–3500 cm^−1^ in all the spectra, a wide range of bands is visible, corresponding to stretching vibrations of –OH bonds present in PVA, SA and the *Echinacea purpurea* extract (active substances including echinacoside and also H-bonded alcohols and phenols) [28,50,51,52]. In the case of the hydrogels obtained by chemical crosslinking, we notice an intense band originating from stretching vibrations of the C-H groups (3000–2850 cm^−1^), stretching vibrations of the –CH_3_ groups (2922 cm^−1^), and vibrations of C-H and O-H bendings at 1350–1330 cm^−1^ [28,29]. This increased intensity is due to the presence of the crosslinking agent—PEGDA. Moreover, when comparing the FT-IR spectra for the samples prepared in the case of chemical crosslinking, it is clearly visible that at the wavenumber about 1750 cm^−1^, it appears vibrations originating from the ester bonds. However, the situation is completely different when it was used ionic crosslinking. Hence, the peak at 1614 cm^−1^ is characteristic for the stretching vibrations of carboxylate anion, which is more intensive in the case of ionic crosslinking, especially for hydrogels modified with *Echinacea purpurea* extract [53]. It can suggest that hydrogels prepared using the chemical method are better crosslinked, which was confirmed the gel fractions results. In addition, the peaks at 1097 and 849 cm^−1^ indicate the presence of the polysaccharide structure [52,53,54].

### 2.4. SEM Analysis

**Figure 7:**
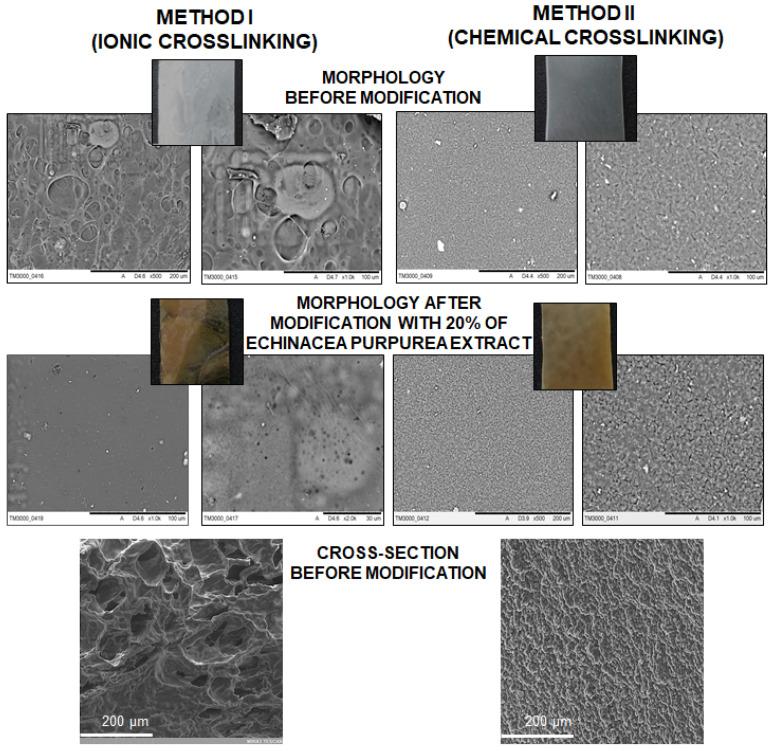
SEM micrographs and photographs of hydrogel materials.

An analysis of SEM micrographs enabled the determination of the surface morphology of the hydrogel materials obtained using the ionic or chemical crosslinking as well as before and after a modification with the *E. purpurea* extract. Additionally, the cross-section of hydrogels was analyzed, which showed significant differences in the structure (Figure 7).

On the basis of the SEM micrographs, it is possible to determine the impact of the preparation method on the morphology. In the case of the samples obtained using chemical crosslinking, the surface is uniform and densely packed. These results are consistent with the literature data [45,55], pointing towards a similar matrix morphology. Moreover, the structure before and after modification is comparable. Additionally, the images in Figure 7 show that the introduction of the *E. purpurea* extract into the matrix caused a color change from milky to light brown. However, the hydrogels obtained by the ionic method have a quite different structures. The surfaces of these materials are more irregular, rough and have bubbles, especially before modification. After the addition of *E. purpurea* extract it can be noticed that the surface has micro- and nanopores. Moreover, the SEM analysis of the cross-section of ionically and chemically crosslinked hydrogels exhibit the significant differences. If the ionic method is used, the structure is more porous and the average pore size is estimated below 100 µm, which impact on the higher swelling degree (especially in PBS) and faster release of active substance. In the case of chemically crosslinked hydrogels, the network is more packed and dense, and the pores cannot be distinguished. Based on the literature, the application of some other preparation routes, such as UV radiation or the freezing/thawing process, makes the surface of a hydrogel more dense or porous [45,56,57]. In summary, a proper selection of the crosslinking method is very important from the point of view of potential applications.

### 2.5. The Release Profile of Echinocoside from SA/PVA/EP Hydrogels

**Figure 8:**
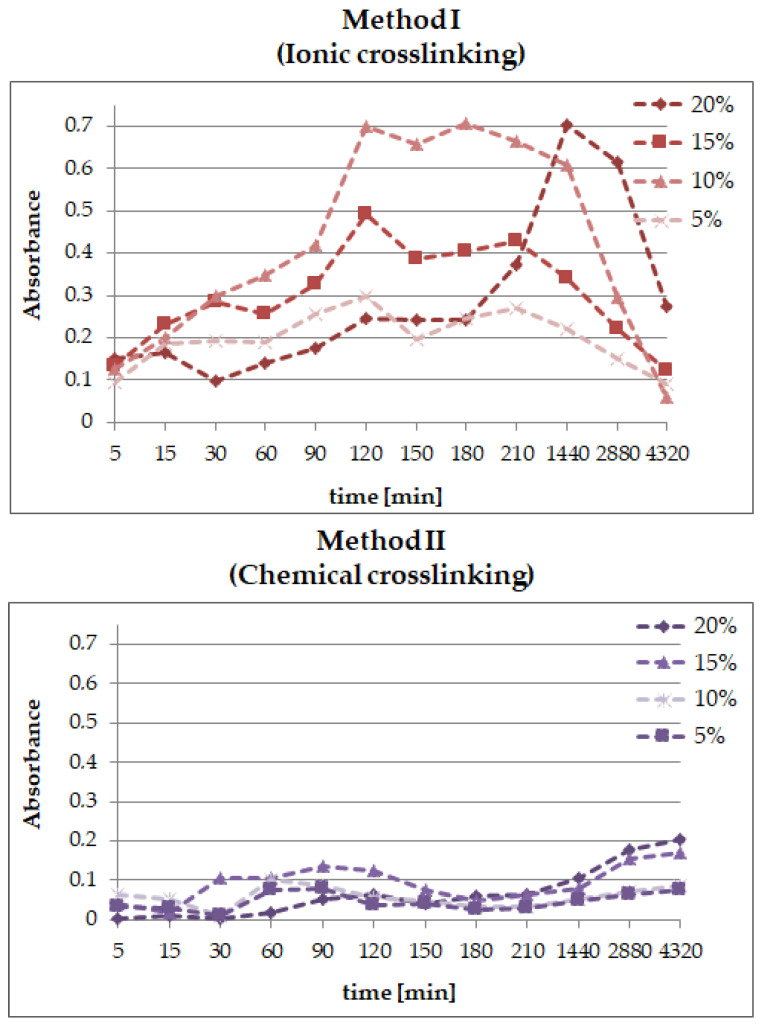
Release profiles of echinacoside from hydrogels prepared using method I (ionic crosslinking) or method II (chemical crosslinking).

The differences in the release profiles of echinacoside from the hydrogels modified by various amounts of the *Echinacea purpurea* extract and obtained using method I—ionic—or method II—chemical—crosslinking are presented in Figure 8.

Generally, it can be noticed that the release profiles of echinacoside from the hydrogels prepared using ionic and chemical crosslinking are completely different. For the samples obtained by ionic crosslinking, the release of the active substance due to rapid surface desorption, the so-called “burst effect”, is observed in the first stage—the first 120 min of the measurement. However, in the case of the samples containing 20% of *E. purpurea* extract, the highest concentration of echinacoside was observed after 24 h. Yet, the “burst effect” does not occur with a lower active ingredient content such as 5% of *E. purpurea* extract. Probably, the concentration is too low. In the next step, absorption of the active substance and its release occur due to hydrogel matrix swelling. During the swelling process, the hydrogel can absorb the extract of *E. purpurea* by volume and, when the system reaches equilibrium, the active substance is released. Based on the analysis of the active substance release profile from the hydrogel obtained by chemical crosslinking, it can be concluded that echinacoside is released gradually, unlike in the materials obtained by the ion crosslinking method. This differences between the release profiles results directly from the structure and porosity of obtained matrix. The ionically crosslinked hydrogels are characterized by a more porous network, which makes that the swelling occurs faster in the PBS environment and the absorption degree is higher compared to the chemically crosslinked matrix. Hence, the active substance released much faster from hydrogel prepared using ionic crosslinking agent.

One may notice a sort of pulsative manner of the release, especially after 30 and 180 min, which is caused by the structure of polymeric matrix and its swelling properties. These results were compared with the release profile of echinacoside from hydrogel materials prepared using UV radiation [45], where the active substance was delivered even for 150 h, but the concentration was very low. That is why it has been concluded that the type of crosslinking method has a significant impact on the release profile of active compounds. Thus, we can design and create new polymeric materials tailored to specific applications.

## 3. Experimental Part

### 3.1. Materials

Sodium alginate and poly(ethylene glycol) diacrylate (PEGDA) M_n_ = 700 g/mol (used as a crosslinking agent) were purchased from Sigma-Aldrich (Steinheim, Germany). Poly(vinyl alcohol) (M_n_ = 72,000 g/mol), ammonium persulphate was employed as an initiator, calcium chloride and glycerine were acquired from POCH SA (Gliwice, Poland). *Echinacea purpurea* herb was purchased from a herbal store—Flos, Mokrsko, Poland. All the chemicals applied in the research were of analytical reagent grade and were used without any further purification. 

### 3.2. Preparation of E. purpurea Extract

An amount of 10 g of weighed, dried and crushed *E. purpurea* was used to prepare water extracts. The extractions were carried out in a Soxhlet apparatus for 4 h with 150 mL of distilled water at the solvent boiling point. The extracts were next stored at 4 °C and used within 1 week. The characterization of the *E. purpurea* extract was described in a previous manuscript [45].

### 3.3. Preparation of SA/PVA/Echinacea Purpurea Hydrogels

**Figure 9:**
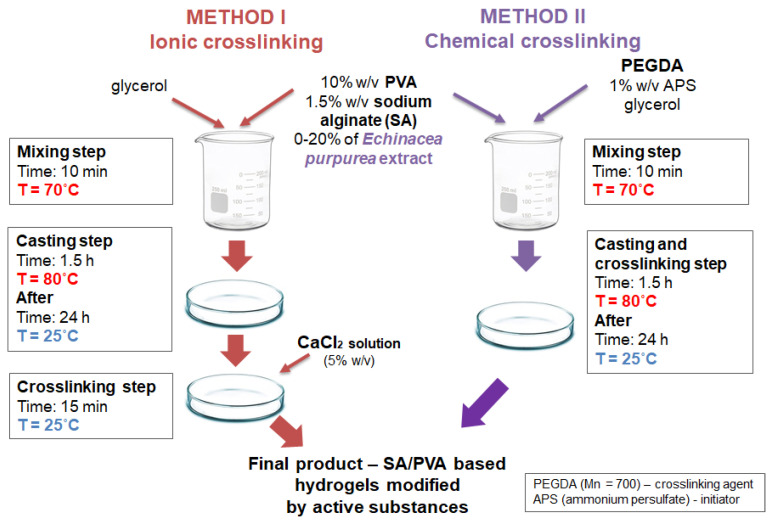
Scheme of preparation procedure.

To obtain SA/PVA/*EP* hydrogels, a 10% (*w*/*v*) solution of poly(vinyl alcohol), 1.5% (*w*/*v*) solution of sodium alginate, *E. purpurea* extract, 1% (*w*/*v*) solution of ammonium persulfate, and 5% (*w*/*v*) solution of calcium chloride were prepared using water. Next, appropriate amounts of these solutions as well as a constant amount of glycerine (3.5%) (using method I—ionic crosslinking) were mixed. In the case of method II—chemical crosslinking—constant amounts of poly(ethylene glycol) diacrylate (7.75%) were added [49,50]. Detailed descriptions of the mixture compositions used for hydrogel preparation are shown in Table 1. Subsequently, the mixtures were heated to 70 °C and 4.4% (*v*/*v*) of ammonium persulfate were added. After that, all the specimens were poured into Petri dishes and placed on a heating plate at a temperature of 80 °C for 1.5 h. In this way, a series of hydrogels with different amounts of the *E. purpurea* extract (5, 10, 15, 20 and 25%, *v*/*v*) were prepared. Finally, the materials were placed for 24 h in ambient conditions. In the next step, in the case of method I, a 5% (*w*/*v*) solution of calcium chloride was added into each sample. The whole procedure is presented in Figure 9 and the compositions of the hydrogels in Table 1.

### 3.4. Determination of Swelling Abilities

The swelling ratio was determined by immersion in distilled water (pH = 6.75), Ringer’s solution (pH = 6.25) and PBS (phosphate-buffered saline, pH = 7.4) at ambient temperature. The dried and weighed (*W_d_*) hydrogel samples were immersed in the fluids. The swollen samples were taken out and weighed (*W_s_*) at specific time intervals after removing the surface water using filter paper. The swelling ratio of all the tested hydrogel samples was determined using the following equation [45,58]:

(1) $$\%SR=\frac{W_{s}-W_{d}}{W_{d}}\cdot 100\%$$

### 3.5. Degradation Test 

An amount of 250 mg of each sample was placed in 50 mL of distilled water (pH = 6.75), Ringer’s solution (pH = 6.25) and PBS (phosphate-buffered saline, pH = 7.4), respectively. The analyzed samples were subsequently placed in an incubator at 37 °C. At specific time points, the pH and conductivity values were determined. The studies were conducted for 28 days at ambient temperature and at 37 °C, every two or three days [45].

### 3.6. ATR-FT-IR

The chemical structure of newly synthesized the hydrogels was confirmed using infrared spectroscopy. The analyses were carried out with a Thermo Scientific Nicolet iS5 FT-IR spectrometer (Thermo Scientific, Madison, WI, USA) equipped with an iD7 ATR accessory in the range of 4000–400 cm^−1^. Spectra were collected at room temperature in a dry atmosphere with a resolution of 2 cm^−1^, and were an average of 20 repeated scans, as the quantity recommended by the manufacturer.

### 3.7. SEM Analysis

The hydrogel morphology was observed using a SEM (Scanning Electron Microscope, Hitachi, Tokyo, Japan) using a Tabletop Microscope TM3000 developed by Hitachi equipped with a low-vacuum backscattered detector and tungsten electron emission at an acceleration voltage of 5.0 kV. The samples were mounted on a specialized holder adjusted in height to optimize operation.

### 3.8. Studies of Echinacoside Release

The *E. purpurea* extract is very rich in many active substances, such as glycosides, flavonoids, polysaccharides, polyacetylenes, pyrrolizidine alkaloids, alkylamines and alkylamides. However, based on the previous research [45], echinacoside is the best substance to determine due to its highest concentration in the extract. That is why the echinacoside release profile from the polymeric matrix in phosphate-buffered saline (PBS, pH = 7.4) at 37 °C was studied in our work. Each release experiment was performed for 3 days. The presence of the drug was confirmed by measuring the absorbance at 360 nm using a UV/Vis spectrophotometer (Evolution 220, ThermoScientific, Waltham, MA, USA).

## 4. Conclusions

The application of different crosslinking methods, even though the hydrogel compositions are the same, has a direct impact on the properties of the final materials:

- the type of the crosslinking method strongly influences the ability of the materials to absorb simulated fluids;
- an increase of the amount of the *Echinacea purpurea* extract caused an increase of the swelling ability of the modified materials regardless of the crosslinking method used;
- the highest degree of swelling was observed in the PBS fluid, after 24 h, but only in the case of the hydrogels prepared through ionic crosslinking;
- the results of the incubation investigations suggest that the modified hydrogels can degrade faster, especially after ionic crosslinking;
- the surface of the hydrogels synthesized using chemical crosslinking is more regular and dense in comparison to the materials prepared using the second method; SEM analysis of the cross-section of hydrogels allow to conclude that the type of crosslinking agents influence directly on their network; in the case of ionically crosslinked hydrogels, the structure is more porous due to the release of echinacoside occurred faster;
- the release profiles show that echinacoside was delivered in a controlled manner for an extended time, but only in the case of the alginate-based hydrogels prepared using chemical crosslinking; however, in the second method, an undesirable phenomenon called the “burst effect” was observed.

## Figures and Tables

**Table 1:** Compositions of SA/PVA/*EP* hydrogels.

**Method I (Ionic Crosslinking)**				
**PVA:A Ratio** **[*v*/*v*]**	**Glycerol** **[%, *v*/*v*]**	***Echinacea purpurea*** **Extract [%, *v*/*v*]**	**CaCl_2_** **[%, *v*/*v*]**	
1:2	3.5	0	35	
		5		
		10		
		15		
		20		
**Method II (Chemical Crosslinking)**				
**PVA:A Ratio** **[*v*/*v*]**	**Glycerol** **[%, *v*/*v*]**	***Echinacea purpurea*** **Extract [%, *v*/*v*]**	**PEGDA** **[%, *v*/*v*]**	**APS** **[%, *v*/*v*]**
1:2	3.5	0	7.75	4.3
		5		
		10		
		15		
		20		

## Data Availability

Not applicable.

## Sample Availability

Samples of the compounds—modified hydrogels available from the authors.
